# Exposure to Environmental Tobacco Smoke and Cognitive Abilities among U.S. Children and Adolescents

**DOI:** 10.1289/ehp.7210

**Published:** 2004-10-07

**Authors:** Kimberly Yolton, Kim Dietrich, Peggy Auinger, Bruce P. Lanphear, Richard Hornung

**Affiliations:** ^1^Cincinnati Children’s Environmental Health Center, Department of Pediatrics, Cincinnati Children’s Hospital Medical Center, Cincinnati, Ohio, USA; ^2^Department of Pediatrics and; ^3^Department of Environmental Health, University of Cincinnati College of Medicine, Cincinnati, Ohio, USA; ^4^Department of Pediatrics, University of Rochester School of Medicine and the American Academy of Pediatrics Center for Child Health Research, Rochester, New York, USA; ^5^Institute for Health Policy and Health Services Research, University of Cincinnati, Cincinnati, Ohio, USA

**Keywords:** children, cognition, environment, environmental tobacco smoke, epidemiology

## Abstract

We used the Third National Health and Nutrition Examination Survey (NHANES III), conducted from 1988 to 1994, to investigate the relationship between environmental tobacco smoke (ETS) exposure and cognitive abilities among U.S. children and adolescents 6–16 years of age. Serum cotinine was used as a biomarker of ETS exposure. Children were included in the sample if their serum cotinine levels were ≤15 ng/mL, a level consistent with ETS exposure, and if they denied using any tobacco products in the previous 5 days. Cognitive and academic abilities were assessed using the reading and math subtests of the Wide Range Achievement Test–Revised and the block design and digit span subtests of the Wechsler Intelligence Scale for Children–III. Analyses were conducted using SUDAAN software. Of the 5,365 6- to 16-year-olds included in NHANES III, 4,399 (82%) were included in this analysis. The geometric mean serum cotinine level was 0.23 ng/mL (range, 0.035–15 ng/mL); 80% of subjects had levels < 1 ng/mL. After adjustment for sex, race, region, poverty, parent education and marital status, ferritin, and blood lead concentration, there was a significant inverse relationship between serum cotinine and scores on reading (β= −2.69, *p* = 0.001), math (β= −1.93, *p* = 0.01), and block design (β= −0.55, *p* < 0.001) but not digit span (β= −0.08, *p* = 0.52). The estimated ETS-associated decrement in cognitive test scores was greater at lower cotinine levels. A log-linear analysis was selected as the best fit to characterize the increased slope in cognitive deficits at lower levels of exposure. These data, which indicate an inverse association between ETS exposure and cognitive deficits among children even at extremely low levels of exposure, support policy to further restrict children’s exposure.

Despite extensive evidence that environmental tobacco smoke (ETS) is associated with an increased risk of detrimental health effects, > 40% of U.S. children are exposed to ETS in their homes (Pirkle et al. 1996). Exposure to ETS has consistently been linked with adverse health effects in children, including middle ear disease (Cook and Strachan 1999), colic (Reijneveld et al. 2000), sudden infant death syndrome (McMartin et al. 2002; Mitchell et al. 1993; Schoendorf and Kiely 1992; Wisborg et al. 2000), asthma exacerbations (Chilmonczyk et al. 1993; Ehrlich et al. 1996; Martinez et al. 1992), and various respiratory difficulties (Cook and Strachan 1999; Gergen et al. 1998; Mannino et al. 2001; Martinez et al. 1988; Rylander et al. 1995). There is increasing but inconsistent evidence that tobacco smoke exposure is linked with intellectual impairments and behavioral problems in children.

Tobacco smoke exposure has been linked to a variety of behavioral (Eskenazi and Castorina 1999; Fried et al. 1992; Orlebeke et al. 1999; Wakschlag et al. 1997; Wasserman et al. 2001; Williams et al. 1998) and developmental (Byrd and Weitzman 1994; Drews et al. 1996; Fried and Watkinson 2000; Fried et al. 1997, 1998; Makin et al. 1991; Olds et al. 1994; Rush and Callahan 1989) consequences for children. Associations with cognitive and achievement problems such as early grade retention (Byrd and Weitzman 1994), reduced vocabulary and reasoning abilities (Eskenazi and Bergmann 1995), and cognitive and intellectual deficits among children (Bauman et al. 1991; Johnson et al. 1999) have also been reported. Still, questions about the role of ETS exposure remain (Eskenazi and Castorina 1999).

Various methodologic limitations of prior studies contribute to the lack of clarity in the findings. Previous research on the effects of tobacco smoke on child outcomes has been limited by small to moderate sample sizes and reliance on parental reports of child exposure. Reports of ETS exposure are complicated by poor recall, an inattention to crucial details such as adjustment for the amount of tobacco exposure, the child’s proximity to the smoker, room ventilation, and other factors that may compromise the validity of exposure measures (Matt et al. 2000). It is difficult to distinguish between effects of prenatal and postnatal tobacco smoke exposure because children who are exposed prenatally also tend to be exposed postnatally. Studies with large sample sizes are therefore needed to separate the independent effects of prenatal and postnatal exposure.

The purpose of this study was to investigate the impact of ETS exposure on children’s cognitive skills, using a large, nationally representative sample of children and adolescents, using serum cotinine, a biomarker of ETS exposure.

## Materials and Methods

The data source for this analysis, the Third National Health and Nutrition Examination Survey (NHANES III), conducted from 1988 to 1994 (Ezzati et al. 1992; National Center for Health Statistics 1994), was a cross-sectional, household survey of the civilian, noninstitutionalized U.S. population. Participant enrollment employed a stratified, multistage, probability sampling design. Data collection included parent and child interviews, direct assessment, health evaluation, and collection of biologic samples in participants’ homes and in a mobile examination center. The present study included data on all eligible children and adolescents 6–16 years of age.

The primary analysis relied on serum cotinine as a measure of ETS exposure. Cotinine, a metabolite of nicotine, can be measured in a number of bodily fluids, as well as in hair, and is the best currently available biomarker of exposure to ETS (Benowitz 1996). We measured serum cotinine with an isotope dilution, liquid chromatography, tandem mass spectrometry method developed and conducted by the National Center for Environmental Health, Centers for Disease Control (Bernert et al. 1997). This method has a reported detection limit of 0.05 ng/mL cotinine. Cotinine values below the limit of detection (left censored data) were imputed by randomly sampling values from the left tail of a log-normal distribution. Results from the imputed method are reported in this article.

Participants were administered two subtests of the Wide Range Achievement Test–Revised (WRAT-R) (Jastak and Wilkinson 1984). The reading subtest assessed letter recognition and word reading, and the math subtest contained oral and written problems ranging from simple addition to calculus. Two subtests from the Wechsler Intelligence Scale for Children–III (WISC-III) (Wechsler 1991) were also administered. The block design subtest assessed visual construction abilities using a set of modeled three-dimensional or printed two-dimensional geometric patterns that the child replicated using a set of red and white cubes. The digit span subtest assessed short-term and working memory by asking the child to repeat a series of increasingly long number sequences forward and backward.

Trained examiners administered tests in a standardized environment using uniform procedures. Ninety-five percent of the children were tested in English; the rest were tested in Spanish. Throughout the study, adherence to the standardized assessment protocol was maintained. Scores on the WRAT-R subtests were standardized to a mean ± SD of 100 ± 15. Scores on the WISC-III subtests were standardized to a mean of 10 ± 3. Appropriate age-standardized scores were used in all analyses.

Children with complete cognitive tests and serum cotinine values were included in the sample. Serum cotinine levels ≤15 ng/mL were used to identify the sample of children exposed to ETS but who were not active smokers, as in a previous NHANES analysis on ETS exposure and child health outcomes (Pirkle et al. 1996). Children were also excluded if they reported using tobacco products in the 5 days before cognitive assessment and blood collection regardless of cotinine level.

### Statistical methods.

All analyses were performed using the SUDAAN statistical package (Shah et al. 1997) to account for the complex sampling design. Appropriate sample weights were applied according to the National Center for Health Statistics guidelines [Centers for Disease Control and Prevention (CDC) 1996] to produce accurate national estimates adjusting for the oversampling of specific population groups within NHANES III. Regression diagnostics were carried out to ensure that results did not depend on influential points, and correlation analyses confirmed lack of collinearity among variables included in the regression models.

Preliminary analyses revealed a nonlinear relationship between mean cognitive scores by various cotinine thresholds. Therefore, cotinine values were log-transformed for analyses to better represent the steeper increase in cognitive scores at lower cotinine values. Analyses using nontransformed cotinine values were performed separately by cotinine values above and below 1 ng/mL (~ 80th percentile) to calculate the linear slopes for these two ranges and test whether there was a significant difference in slope.

We calculated geometric mean serum cotinine concentrations and mean cognitive test scores for potential covariates based on a review of the literature. These variables included sex, race and ethnicity, poverty index (based on a ratio of family income and family size), parent educational level and marital status, region of the country, ferritin as a measure of iron status, and blood lead level. Poverty index, ferritin, and lead levels were categorized into terciles based on the sample distribution.

We investigated the effects of potential confounding factors identified from bivariate analyses by multiple linear regression analyses with log-transformed serum cotinine (nanograms per milliliter) concentration treated as a continuous independent variable. Graphical displays of the independent relationship between log-transformed cotinine and each cognitive outcome were generated. We also calculated adjusted mean cognitive scores for various levels of cotinine thresholds.

Parental interview data on prenatal exposure to tobacco smoke, birth weight, and history of neonatal intensive care unit (NICU) stay were available for a subsample of children 6–11 years of age. We conducted a secondary analysis to verify that inclusion of perinatal variables available only for this subsample did not alter findings of the larger sample.

## Results

Of the 5,683 children and adolescents in NHANES III who were 6–16 years of age, 4,619 (81.3%) completed at least one test of cognitive abilities and had available serum cotinine values. Children with serum cotinine levels > 15 ng/mL (*n* = 155) or who reported active smoking during the 5 days before testing (*n* = 65) were excluded from this analysis. This yielded a final sample of 4,399 children (77.4% of all 6- to 16-year-olds) in the primary analysis. Children who were excluded from the final sample had lower scores on tests of math (*p* < 0.001), reading (*p* = 0.003), and block design (*p* = 0.02) than did children in the analysis. Excluded children were also more likely to live in households with lower marriage rates (*p* = 0.02). Because of our eligibility criteria, excluded children also had significantly higher levels of serum cotinine (*p* < 0.001).

Serum cotinine was detectable in 84% of children in the final sample, with 16% having cotinine levels below the limit of detection (< 0.05 ng/mL). After imputing randomly selected values from the left tail of a log-normal distribution, the geometric mean serum cotinine level for the sample was 0.23 ng/mL (SE = 0.01). Serum cotinine concentrations varied by children’s characteristics (Table 1). Serum cotinine concentrations were significantly higher among African Americans than Hispanics or non-Hispanic whites, among children of parents with a lower household income or lower educational achievement, among children living in the Midwest United States, and among those with higher blood lead concentrations. Children exposed to both prenatal and postnatal smoke and those exposed to postnatal smoke alone also had higher serum cotinine levels.

Consistent with previous research (Jordaan et al. 1999), mean serum cotinine levels were significantly higher among children who had at least one smoker living in their home (*p* < 0.001). Children’s serum cotinine levels also increased as the number of smokers in a household increased (*p* < 0.001) and as the number of cigarette packs smoked per day in a household increased (*p* < 0.001).

Overall mean scores for math, reading, block design, and digit span are presented in Table 1. In unadjusted analyses, cognitive performance scores differed significantly by sex, race or ethnicity, poverty status, parent marital status and educational level, and blood lead concentration. There was also a significant inverse relationship between serum cotinine and cognitive test scores. Children with the highest serum cotinine levels received significantly lower performance scores on all four tests than did children in the lowest cotinine level.

In multiple regression analyses using the log-transformation of cotinine and adjusting for covariates, serum cotinine was significantly associated with lower scores for reading, math, and visuospatial skills (Table 2). An increase in the log serum cotinine from level 1 to 10 ng/mL was associated with a 1.93-point loss in math scores (*p* ≤0.001) and a 2.7-point loss in reading scores (*p* ≤0.001) for tests with a standardized mean of 100. The same change in the log serum cotinine level was associated with a 0.55-point loss in block design scores (*p* ≤0.001) and a 0.08-point loss in digit span scores for tests with a standardized mean of 10.

There was a significant inverse relationship between the log of serum cotinine and cognitive abilities at lower levels of exposure. Children with serum cotinine levels < 0.1 ng/mL had an adjusted average reading score of 94.7. Children with cotinine levels between 0.1 and 1 ng/mL had an average 2.6-point drop in reading scores, children with levels 1–3 ng/mL had an additional 0.2-point drop in reading, and children with cotinine values > 3 ng/mL had an additional 4.8-point drop in reading score. Although the greatest decrease in reading scores was observed among children with higher cotinine levels (range, 3–15 ng/mL), there was a greater proportional change in reading scores per unit of cotinine exposure at levels in the range of 0.1–1 ng/mL. According to population estimates employing the appropriate sampling weights, we estimated that > 33.3 million children are at risk for ETS-related reading deficits (i.e., children with cotinine levels ≤15 ng/mL). Math and block design scores showed similar trends in decreasing cognitive scores with increasing cotinine levels (Table 3).

Questionnaire data on maternal smoking during pregnancy, birth weight, and NICU stay were available for a subsample of 2,738 children 6–11 years of age. Among this sub-sample of children, the covariate-adjusted relationship between the log of serum cotinine and cognitive scores indicates that an increase in the log serum cotinine from 1 to 10 ng/mL was associated with a related 2.4-point decrease in reading scores (*p* ≤0.05), a 1.6-point decrease in math scores, and a 0.42-point decrease in block design scores (*p* ≤0.05). In secondary analyses, inclusion of prenatal tobacco smoke exposure, birth weight, and NICU stay had little effect on the relationship between ETS exposure and reading scores (*p* ≤0.05). In contrast, the association of ETS exposure with block design was attenuated (Table 4).

**Figure 1:**
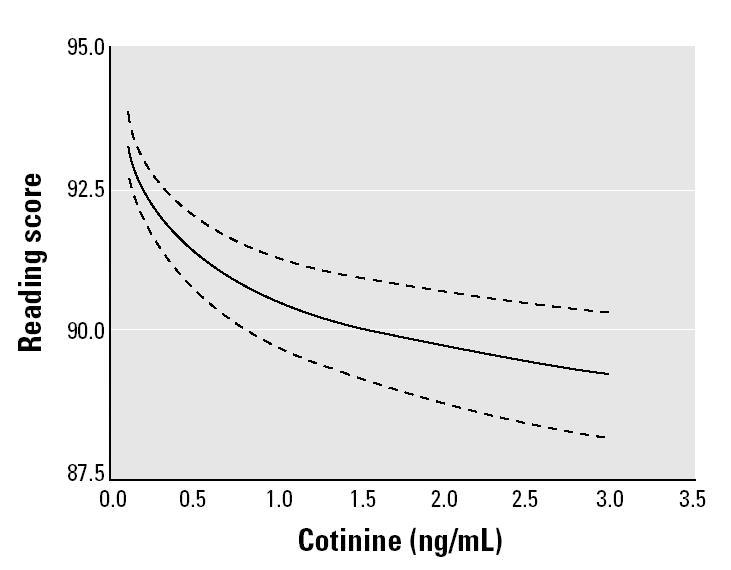
Log-linear regression line for reading scores by serum cotinine levels. Dashed lines indicate 95% confidence interval.

**Figure 2:**
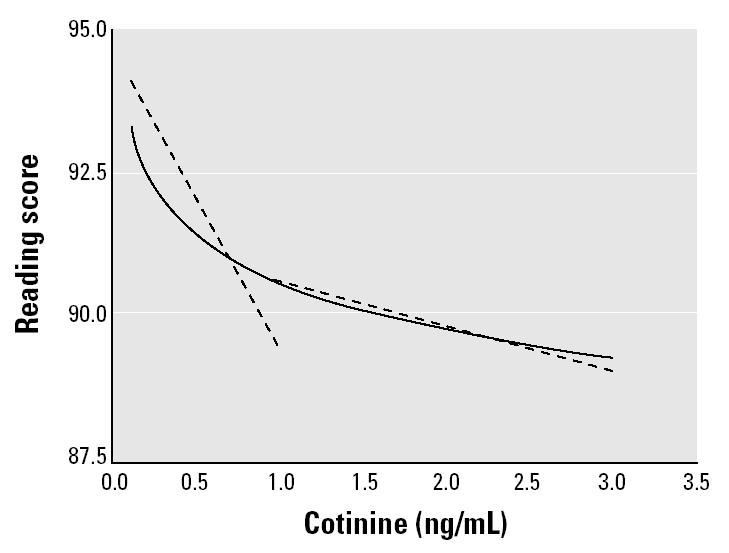
Log-linear model for cotinine (solid line) versus linear models for cotinine among children with cotinine above and below 1 ng/mL (dashed lines; ~ 80th percentile).

The ETS-associated decrements in reading scores appeared to be greater at lower levels of serum cotinine (Figure 1). Math and block design also showed a steeper decline in scores at lower cotinine values than at higher values. To test whether the difference in the slopes was statistically significant, we conducted a stratified adjusted analysis including linear (non-transformed) cotinine values above and below 1 ng/mL (~80th percentile). Children with cotinine values ≤1 ng/mL (< 80th percentile) had an average 5.0-point decrease in reading scores for each 1-ng/mL increase in serum cotinine compared with an average 0.8-point decrease in reading scores for children with cotinine values above 1 ng/mL (> 80th percentile) (Figure 2). The difference between these averages was statistically significant (*t* = 2.38, *p* = 0.02).

## Discussion

In this study we used serum cotinine, a bio-marker of ETS exposure, to examine the relationship between ETS exposure and cognitive abilities in a large, nationally representative sample of children and adolescents. A dose–response relationship was found in which higher levels of ETS exposure were associated with greater deficits in reading, math, and visuospatial reasoning but not short-term memory. The inverse relationship persisted at extremely low levels of exposure. Indeed, the estimated decrement appeared to be greater at lower serum cotinine levels. These data, in combination with other experimental and human studies linking ETS exposure with decreased performance in tests of reasoning ability and language development (Bauman et al. 1991; Eskenazi and Bergmann 1995) and tests of intelligence (Johnson et al. 1999) and an increased risk for grade retention (Byrd and Weitzman 1994) suggest that ETS may be causally associated with impairments in cognitive skills.

Reading ability was especially sensitive to ETS exposure. We observed a significant inverse ETS-associated decrement in reading scores that persisted at levels of ETS exposure < 0.5 ng/mL. We also found that the decrements in reading scores were greater in magnitude at the lowest levels of ETS exposure. Similarly, tobacco exposure during pregnancy has an effect on infant birth weight that demonstrates a steeper slope at lower levels of exposure (England et al. 2001). A similar phenomenon has been observed recently in the area of lead research, where children with blood lead levels < 10 μg/dL experience greater decrements in cognitive abilities for each 1-μg/dL increase in blood lead compared with children with higher blood lead levels (Canfield et al. 2003; Lanphear et al. 2000). The reason for the strikingly large decrement in reading scores and birth weight at lower serum cotinine levels is unclear and needs to be carefully evaluated in prospective studies including more frequent measures of biomarkers of exposure.

The relationship between tobacco smoke exposure and childhood reading skills has been previously explored. Researchers found links between postnatal ETS exposure and decrements in receptive vocabulary (Bauman et al. 1991; Eskenazi and Bergmann 1995). In a longitudinal study by Fried et al. (1997), a significant negative dose–response relationship with prenatal tobacco smoke exposure and specific reading functions was reported. Exposure to tobacco smoke products *in utero* was related to deficits in children’s abilities to use contextual cues and comprehension skills in understanding written passages. In this instance, reading was more seriously affected in the broad sense of understanding words in context rather than understanding individual words. Because the NHANES III used an instrument to measure letter recognition and word reading, we are unable to specifically test reading comprehension; however, children who have difficulties recognizing and reading individual words are also likely to have problems with comprehension.

We also found an inverse relationship between ETS exposure and visuospatial reasoning skills. The ETS-associated impairment in visuospatial skills persisted to lower levels of exposure, increasing in magnitude among children who had the lowest levels of exposure. This finding supports previous studies that found children exposed to ETS during childhood performed significantly more poorly on reasoning tasks compared with children who were either unexposed to tobacco smoke or exposed during the prenatal period only (Bauman et al. 1991; Eskenazi and Bergmann 1995). In a more detailed investigation of tobacco smoke’s impact on visuoperceptual skills, Fried and Watkinson (2000) found that children exposed to tobacco smoke prenatally experienced a great deal of difficulty in aspects of visual discrimination, visual memory, and visual–spatial relationships. It is plausible that a similar effect may arise from postnatal ETS exposure, but this will require further investigation.

The negative association between ETS exposure and math skills found in this study was highly significant. It did not, however, persist at the lowest levels of exposure in our sample. The overall log-linear regression line nonetheless suggested a general decrease in math scores as cotinine levels rise. Fried et al. (1998) found no relationship between prenatal tobacco smoke exposure and math skills. Other researchers have reported a positive effect of tobacco smoke on alertness and cognitive function among the elderly or impaired as well as in animal studies (Picciotto and Zoli 2002). Additional studies are needed to validate our findings that ETS exposure has a negative impact on math skills.

Three main aspects of the present study add strength to the conclusion that ETS exposure is a neurotoxin that is linked with cognitive deficits. First, the large sample size allows for the inclusion of numerous potential covariates while retaining statistical power. Second, this is the first study to rely primarily on a bio-marker of postnatal ETS exposure in examining this association, thus reducing recall bias. Third, our findings are consistent with specific adverse effects observed in other studies, including deficits in reading and visuospatial reasoning skills (Bauman et al. 1991; Eskenazi and Bergmann 1995; Fried and Watkinson 2000; Fried et al. 1997).

This study has some limitations. The NHANES III design did not include measures of cognitive abilities of parents or of the quality of the home environment. Instead, we relied on maternal education, income, and marital status as surrogate markers in our analyses. ETS exposure was assessed through the measurement of serum cotinine, an indicator of exposure within a few days. It is unclear whether short-term exposure (i.e., serum cotinine, which has a half-life of 48–72 hr) is representative of the child’s chronic exposure or is indicative of the short-term toxicity of ETS exposure. Other studies have found serum cotinine levels to be stable over time among smokers and nonsmokers (de Leon et al. 2002; Kemmeren et al. 1994). In future studies, markers of both short-term and long-term exposure should be evaluated with cognitive outcomes, and further exploration of potential mechanisms underlying these effects is needed.

One of the difficulties in performing research on childhood ETS exposure is the challenge of distinguishing the effects of prenatal tobacco smoke exposure from childhood (postnatal) ETS exposure. Because children exposed to ETS are often those who were also exposed to tobacco smoke prenatally, large sample sizes are necessary to distinguish any adverse effects of prenatal ETS exposure from postnatal ETS exposure. Among the subsample of 6- to 11-year-olds with prenatal data, 23.6% of children had been exposed to tobacco smoke during pregnancy. This is equivalent to the current adult smoking rate in the United States (CDC 2001) and twice the reported rate of smoking during pregnancy when the NHANES III data were being collected (Matthews 2001). Nevertheless, the significant negative association between ETS exposure and reading ability persisted even when the prenatal variables of exposure to tobacco smoke, birth weight, and NICU stay were included among this subsample. These prenatal data were obtained by parental report and may result in an underestimate of the intensity of tobacco smoke exposure. To confirm the causal role of ETS in diminished cognitive abilities among children, prospective birth cohort studies will be necessary.

The mechanisms by which ETS may exert its effects on cognitive function are unknown. Research into the effects of nicotine and cotinine (Audesirk and Cabell 1999) on neurite length suggest that exposure to these substances during prenatal development, as with lead exposure (Schneider et al. 2003), may affect both the survival and growth of essential nervous system components even at very low levels of exposure. Although prenatal exposure to tobacco smoke has been found to affect neurite growth and neuronal connections, more research is needed to explore the mechanism by which postnatal ETS affects cognitive ability and whether this is a similar or different mechanism from the effects during prenatal development.

ETS is recognized as a serious threat to public health. Still, acceptable levels of exposure have not been established. From the present analysis, we are unable to recommend a safe level of exposure to ETS because there is no discernable threshold for the impact of ETS on the cognitive functioning of children. It is likely that further analyses of the data being acquired through the ongoing NHANES will provide an opportunity to explore ETS-related impairments at even lower serum cotinine levels because ETS exposure has declined significantly over the past decade (CDC 2002).

## Conclusion

The findings of this study confirm previous research indicating an inverse relationship with ETS exposure and cognitive outcomes. We also provide new information indicating that ETS is neurotoxic at extremely low levels. Exposure to ETS in U.S. children therefore has substantial public health impact beyond asthma, otitis media, and other widely recognized adverse consequences. According to population estimates employing the appropriate sampling weights, we estimated that > 21.9 million children are at risk for ETS-related reading deficits. Although further research is necessary to confirm these findings, this analysis along with other studies provides adequate evidence to support policy to further reduce childhood exposure to ETS.

## Figures and Tables

**Table 1:** Mean serum cotinine concentrations and cognitive test scores for children and adolescents, 6–16 years of age, NHANES III (1988–1994).

Variable	Geometric mean serum cotinine level (ng/mL)	Math	Reading	Block design	Digit span
Total (*n* = 4,399)	0.23	94.6	92.5	9.6	8.7
Sex					
Male	0.22	94.2	92.1	10.0*^##^*	8.6*
Female	0.24	95.0	92.9	9.2*^##^*	8.8*
Race/ethnicity					
Non-Hispanic black	0.45*^##^*	86.5*^##^*	84.9*^##^*	7.1*^##^*	8.0*^##^*
Non-Hispanic white (referent)	0.22	97.6	95.6	10.3	9.1
Hispanic	0.14*^#^*	87.6*^##^*	86.3*^##^*	8.7*^##^*	7.5*^##^*
Non-Hispanic other	0.16	99.5	90.4	10.2	8.2**
Region					
Midwest	0.32*	96.0	93.2	10.1	9.0*
South	0.28	93.0	91.8	8.9*	8.5
West	0.15	95.8	92.0	10.1*	8.7
Northeast (referent)	0.20	94.3	93.7	9.6	8.6
Parent marital status					
Married	0.20*^##^*	96.1*^##^*	93.6*^##^*	9.8*^##^*	8.8*^##^*
Not married	0.41*^##^*	88.9*^##^*	88.1*^##^*	8.7*^##^*	8.2*^##^*
Parent education level					
< High school graduate	0.39*^##^*	86.2*^##^*	83.6*^##^*	8.3*^##^*	7.7*^##^*
High school graduate	0.30*^##^*	92.8*^##^*	91.1*^##^*	9.2*^##^*	8.5*^##^*
> High school graduate (referent)	0.14	100.5	98.1	10.6	9.5
Poverty index ratio					
Lower tercile	0.37*^##^*	87.8*^##^*	84.8*^##^*	8.4*^##^*	8.0*^##^*
Middle tercile	0.22*	94.3*^##^*	92.5*^##^*	9.5*^##^*	8.6*^##^*
Higher tercile (referent)	0.15	101.2	99.7	10.8	9.8
Lead					
Lower tercile (referent)	0.16	97.7	96.2	9.9	9.0
Middle tercile	0.24 *^#^*	94.7**	92.3*^##^*	9.8	8.7*
Higher tercile	0.48*^##^*	87.7*^##^*	84.6*^##^*	8.5 *^##^*	8.1*^##^*
Ferritin					
Lower tercile (referent)	0.21	94.5	92.2	9.7	8.7
Middle tercile	0.23	94.9	92.9	9.7	8.8
Higher tercile	0.24	94.6	92.5	9.4	8.7
Smoking*^a^*					
Prenatal and postnatal	1.48*^##^*	92.1**	87.5*^#^*	8.9*^##^*	8.5*
Prenatal only	0.14	92.0	93.9	10.1	8.8
Postnatal only	0.77*^##^*	92.0*^##^*	89.1*^##^*	9.1*^##^*	8.4*^##^*
None (ref)	0.10	96.5	92.6	10.2	9.1
Received care in NICU*^a^*					
Yes	0.30	93.4	89.1	9.9	8.7
No	0.24	94.7	91.4	9.7	8.9
Birth weight*^a^*					
< 2,500 g	0.41*	88.9*^#^*	87.2*	8.2*^##^*	8.1*
> 2,500 g	0.24*	95.0*^#^*	91.6*	9.9*^##^*	9.0*

A significant association is compared with the referent group. For bivariate categories the referent group for one category is the other group.

a Includes only children 6–11 years of age.

* *p* ≤0.05;

** *p* ≤0.01;

# *p* ≤0.005;

## *p* ≤0.001.

**Table 2:** Log-linear effect of cotinine ≤15 ng/mL [β-coefficient (SE β)] and potential covariates on cognitive test scores at 6- to 16 years of age, NHANES III (1988–1994).

	Math	Reading	Block design	Digit span
Log cotinine (ng/mL)	−1.93 (0.70)**	−2.69 (0.75)*^##^*	0.55 (0.12)*^##^*	−0.08 (0.13)
Sex				
Male	−1.09 (0.80)	−0.78 (0.83)	0.76 (0.18)*^##^*	−0.34 (0.13)*
Female (referent)	0.00 (0.00)	0.00 (0.00)	0.00 (0.00)	0.00 (0.00)
Race/ethnicity				
African American	−5.41 (1.05)*^##^*	−4.90 (0.91)*^##^*	−2.26 (0.18)*^##^*	−0.56 (0.17)*^#^*
Hispanic	−4.91 (1.27)*^##^*	−3.89 (1.11)*^##^*	−0.96 (0.29)*^#^*	−0.90 (0.23)*^##^*
Other	4.06 (1.92)*	−0.98 (3.04)	0.22 (0.36)	−0.63 (0.45)
White (referent)	0.00 (0.00)	0.00 (0.00)	0.00 (0.00)	0.00 (0.00)
Parent education	0.90 (0.13)*^##^*	0.87 (0.16)*^##^*	0.14 (0.03)*^##^*	0.15 (0.02)*^##^*
Poverty status	1.81 (0.42)*^##^*	2.25 (0.36)*^##^*	0.33 (0.08)*^##^*	0.20 (0.07)**
Region				
Midwest	2.38 (2.28)	0.61 (2.31)	0.95 (0.23)*^##^*	0.49 (0.23)*
South	0.68 (1.87)	−0.12 (2.30)	−0.01 (0.21)	0.12 (0.23)
West	1.47 (2.35)	−1.56 (2.63)	0.63 (0.22)**	0.30 (0.26)
Northeast (referent)	0.00 (0.00)	0.00 (0.00)	0.00 (0.00)	0.00 (0.00)
Parent marital status				
Not married	−1.62 (1.29)	0.94 (1.03)	0.36 (0.20)	−0.001 (0.16)
Married (referent)	0.00 (0.00)	0.00 (0.00)	0.00 (0.00)	0.00 (0.00)
Blood lead (ng/L)	−0.57 (0.17)*^#^*	−0.80 (0.21)*^##^*	−0.08 (0.03)*	−0.03 (0.02)
Ferritin (ng/L)	0.01 (0.01)	0.01 (0.01)	−0.002 (0.001)	−0.001 (0.001)

* *p* ≤0.05;

** *p* ≤0.01;

# *p* ≤0.005;

## *p* ≤0.001.

**Table 3:** Adjusted mean cognitive test scores (mean ± SE) at increasing log cotinine levels for children 6–16 years of age, controlling for potential covariates, NHANES III (1988–1994).

Cotinine level (ng/mL)	Math	Reading	Block design	Digit span
< 0.1	95.83 ± 0.94	94.67 ± 0.81	9.91 ± 0.15	8.87 ± 0.13
0.1–1	94.69 ± 0.79	92.12 ± 0.82	9.59 ± 0.10	8.67 ± 0.09
1–3	94.72 ± 1.05	91.88 ± 1.34	9.43 ± 0.21	8.87 ± 0.22
3–15	88.69 ± 1.93	87.13 ± 1.93	8.52 ± 0.30	8.00 ± 0.29

**Table 4:** Adjusted slope of log cotinine [β-coefficient (SE β)] on cognitive test scores between the full sample of 6–16-year-olds and subsample of 6–11-year-olds.

	Full sample (*n* = 4,399)	Subsample (*n* = 2,738)	Subsample including prenatal data
Math	−1.93 (0.70)**	−1.63 (0.88)	−1.15 (1.03)
Reading	−2.69 (0.75)^##^	−2.37 (0.93)*	−1.94 (0.87)*
Block design	−0.55 (0.12)^##^	−0.42 (0.16)*	−0.28 (0.16)
Digit span	−0.08 (0.13)	−0.11 (0.16)	−0.07 (0.18)

* *p* ≤0.05;

** *p* ≤0.01;

## *p* ≤0.001.
